# Supplementary material for: Disparities in Medicaid and Medicare physician reimbursements for ophthalmic procedures
Source: PLoS One. 2025 Jun 18;20(6):e0324383. doi: 10.1371/journal.pone.0324383 (PMC12176135; doi:10.1371/journal.pone.0324383)
Supplement: S1 Supplemental Table 1 — (DOCX) [file pone.0324383.s001.docx]

**Supplemental Table 1. Medicare and Medicaid Data Sources**

| **Location** | **Data Source/Website** |
| --- | --- |
| Entire United States | <https://www.cms.gov/medicare/physician-fee-schedule/search/license-agreement?destination=/medicare/physician-fee-schedule/search%3F> |
|  |  |
| Alabama^a^ | <https://medicaid.alabama.gov/content/Gated/7.3G_Fee_Schedules/7.3G_Physician_Fee_Schedule_9-2-20.pdf> |
| Alaska | <http://manuals.medicaidalaska.com/medicaidalaska/providers/FeeSchedule.asp> |
| Arizona | <https://www.azahcccs.gov/PlansProviders/RatesAndBilling/FFS/Physicianrates/> |
| Arkansas | <https://humanservices.arkansas.gov/wp-content/uploads/PHYSICN-fees.pdf> |
| California | <https://files.medi-cal.ca.gov/rates/rates_range_display.aspx> |
| Colorado | <https://hcpf.colorado.gov/sites/hcpf/files/01_CO_Fee%20Schedule_Health%20First%20Colorado_01012021_V1.9.pdf> |
| Connecticut | <https://www.ctdssmap.com/CTPortal/Provider/ProviderFeeScheduleDownload/tabid/54/Default.aspx> |
| Delaware | <https://medicaidpublications.dhss.delaware.gov/docs/DesktopModules/Bring2mind/DMX/API/Entries/Download?Command=Core_Download&EntryId=1218&language=en-US&PortalId=0&TabId=94> |
| D.C. | <https://www.dc-medicaid.com/dcwebportal/nonsecure/feeScheduleInquiry> |
| Florida | <https://ahca.myflorida.com/medicaid/review/Reimbursement/2021-01-01_Fee_Sched_Billing_Codes/Practitioner_Fee_Schedule_2021.pdf> |
| Georgia | <https://www.mmis.georgia.gov/portal/Portals/0/StaticContent/Public/ALL/FEE%20SCHEDULES/Schedule%20of%20Maximum%20Allw%20Pymt%20Physician_%20(004)%2020210422173529.pdf> |
| Hawaii^a^ | <https://medquest.hawaii.gov/content/dam/formsanddocuments/resources/Provider-Resources/fee-schedules/medicaid/Fee_Schedule_20200624_FINAL.pdf> |
| Idaho | <https://publicdocuments.dhw.idaho.gov/WebLink/Browse.aspx?id=15127&dbid=0&repo=PUBLIC-DOCUMENTS> |
| Illinois | <https://www.illinois.gov/hfs/MedicalProviders/MedicaidReimbursement/Pages/Practitioner.aspx> |
| Indiana | <http://provider.indianamedicaid.com/ihcp/Publications/MaxFee/fee_home.asp#OutpatientFeeSchedule> |
| Iowa | <https://secureapp.dhs.state.ia.us/MedicaidFeeSched/> |
| Kentucky | <https://chfs.ky.gov/agencies/dms/DMSFeeRateSchedules/2021PhysicianFeeSchedule.pdf> |
| Louisiana | <https://www.lamedicaid.com/provweb1/fee_schedules/FEESCHED.pdf> |
| Maine^a^ | <https://mainecare.maine.gov/Provider%20Fee%20Schedules/Rate%20Setting/Section%20090%20-%20Physician%20Services/Section%2090%20-%20Physician%20Services%202020.pdf> |
| Maryland | <https://mmcp.health.maryland.gov/Pages/Provider-Information.aspx> |
| Massachusetts | <https://www.mass.gov/doc/rates-for-surgery-and-anesthesia-services-effective-august-1-2021-0/download>  <https://www.mass.gov/doc/vision-care-services-and-ophthalmic-materials-effective-january-1-2020-0/download> |
| Michigan | https://www.michigan.gov/documents/mdhhs/Practitioner-042021_722462_7.pdf |
| Minnesota^a^ | <https://mn.gov/dhs/assets/mhcp-fee-schedule_tcm1053-294225.pdf> |
| Mississippi^a^ | <https://www.ms-medicaid.com/msenvision/feeScheduleInquiry.do> |
| Missouri | <https://apps.dss.mo.gov/fmsFeeSchedules/DLFiles.aspx> |
| Montana | [**https://medicaidprovider.mt.gov/docs/feeschedules/2021FS/January2021FS/January2021PhysicianServicesFeeScheduleV3rev04092021.pdf**](https://medicaidprovider.mt.gov/docs/feeschedules/2021FS/January2021FS/January2021PhysicianServicesFeeScheduleV3rev04092021.pdf) |
| Nebraska^a^ | https://dhhs.ne.gov/Pages/Medicaid-Provider-Rates-and-Fee-Schedules.aspx?Paged=TRUE&p_Fee_x0020_Schedule=Injectables&p_Effective_x0020_Date=20190101%2006%3A00%3A00&p_ID=131&PageFirstRow=91&&View=%7B231DB632-0133-4683-B02E-FE97BDE343E5%7D#InplviewHashd0c735e5-ed55-4b8e-b5e1-056e3fd349dd=Paged%3DTRUE-p_Fee_x0020_Schedule%3DPhysician%2520Services-p_Effective_x0020_Date%3D20190701%252005%253a00%253a00-p_ID%3D190-FolderCTID%3D0x012001-PageFirstRow%3D181 |
| Nevada^a^ | <http://dhcfp.nv.gov/Resources/Rates/FeeSchedules/> |
| New Hampshire | <https://nhmmis.nh.gov/portals/wps/wcm/connect/a7c77d0041c4e06e809c8c869857d373/2021+Fee+Schedule-Covered+Procedures+Report+as+of+01-01-2021.pdf?MOD=AJPERES> |
| New Jersey | https://www.njmmis.com/downloadDocuments/CPTHCPCSCODES2021.pdf |
| New Mexico^a^ | <https://www.hsd.state.nm.us/providers/fee-for-service/> |
| New York | <https://www.emedny.org/ProviderManuals/Physician/index.aspx> |
| North Carolina | <https://files.nc.gov/ncdma/documents/Fee-Schedules/Physicians/Physician-Service-Fee-Schedule-20210322.pdf> |
| North Dakota^a^ | https://www.nd.gov/dhs/services/medicalserv/medicaid/docs/fee-schedules/2020-professional-services-fee-schedule.pdf |
| Ohio^a^ | <https://medicaid.ohio.gov/Portals/0/Providers/FeeScheduleRates/App-DD.pdf> |
| Oklahoma | <https://oklahoma.gov/ohca/providers/claim-tools/fee-schedule.html> |
| Oregon | <https://www.oregon.gov/oha/HSD/OHP/DataReportsDocs/fee-schedule0321.pdf> |
| Pennsylvania | <https://www.humanservices.state.pa.us/OUTPATIENTFEESCHEDULE/Home/> |
| Rhode Island | <https://www.eohhs.ri.gov/sites/g/files/xkgbur226/files/2021-03/Medicaid%20Fee%20Schedule%202021.pdf> |
| South Carolina | <https://www.scdhhs.gov/resource/fee-schedules> |
| South Dakota | <https://dss.sd.gov/docs/medicaid/providers/feeschedules/Physician_Services_current.pdf> |
| Texas | <https://public.tmhp.com/FeeSchedules/OnlineFeeLookup/FeeScheduleSearch.aspx> |
| Utah | <http://health.utah.gov/stplan/lookup/CoverageLookup.php> |
| Vermont | <http://www.vtmedicaid.com/#/feeSchedule/cptCodes> |
| Virginia^a^ | <https://www.dmas.virginia.gov/for-providers/general-information/procedure-fee-files-cpt-codes/> |
| Washington | https://www.hca.wa.gov/billers-providers-partners/prior-authorization-claims-and-billing/provider-billing-guides-and-fee-schedules |
| West Virginia | <https://dhhr.wv.gov/bms/FEES/Documents/RBRVS%20Fee%20Schedules/BMS-2021%20Physician%27s%20(RBRVS)%20FS%20eff%204.1.21-3.31.22.pdf> |
| Wisconsin | <https://www.forwardhealth.wi.gov/WIPortal/Subsystem/Publications/MaxFeeDownload.aspx> |
| Wyoming | <https://wymedicaid.portal.conduent.com/fees/Fee_Schedule/index.asp> |

a: State’s Medicaid Department was emailed or called on the phone to either obtain or confirm updated physician fee schedule for 2021
